# Marginal Adaptation of In Vitro Class II Restorations Made Out of Bulk or Conventional Composite Using Single- or Multi-Layered Techniques

**DOI:** 10.3390/ma16186325

**Published:** 2023-09-21

**Authors:** Didier Dietschi, Mustafa Askari, Isaline Rossier, Luciana Caseiro, Ivo Krejci, Julian Gregoire Leprince, Enrico Di Bella, Stefano Ardu

**Affiliations:** 1 Division of Cariology and Endodontology, Section of Dental Medicine, Faculty of Medicine, University of Geneva, 1205 Geneva, Switzerland; didier.dietschi@unige.ch (D.D.); dr.m.askari@cddo.ch (M.A.); isaline.rossier@unige.ch (I.R.); luciana.nunes@unige.ch (L.C.); ivo.krejci@unige.ch (I.K.); julian.leprince@unige.ch (J.G.L.); 2 Department of Political and International Studies, University of Genoa, 16126 Genoa, Italy; enrico.dibella@unige.it

**Keywords:** bulkfill, marginal adaptation, resin composite, class II

## Abstract

***Objective***: Testing the influence of various restorative materials (conventional or bulkfill composites) and filling techniques (single- or multi-layered techniques) on the in vitro marginal adaptation of large class II direct composites with supra and sub-gingival margins subjected to thermomechanical loading (TML). ***Methods***: A total of 40 prepared teeth were attributed randomly to five experimental groups. In Group 1, restorations were made of multi-layered high-viscosity conventional composite (Tetric EvoCeram); in Groups 2 and 3, restorations were made of a high viscosity bulkfill composite (Tetric EvoCeram Bulk Fill) applied in one (Group 2) or three layers (Group 3); in Groups 4 and 5, restorations were made of a flowable bulkfill composite (SDR flow) applied in one (Group 4) or two layers (Group 5), covered with a layer of high-viscosity conventional composite (Ceram-X Universal). A single adhesive (OptiBond FL) was used in all groups. All specimens were submitted to a staged TML comprising three phases (2 × 500,000 and 1,000,000 cycles) at 50 N with 3350 thermal cycles (5 to 55 °C) every 500,000 cycles. The tooth–restoration interfaces on proximal surfaces were evaluated quantitatively by scanning electron microscopy, before and after each TML phase, hence at three timepoints (T0, T1, T2 and T3). The following segments were considered for evaluation: proximal, vertical enamel margins (assessed individually on both restoration sides), cervical enamel (restoration side above CEJ) and cervical dentin margin (restoration side below the CEJ). ***Results***: TML induced a significant reduction in continuous adaptation at both enamel and dentin margins in all groups, with percentages of continuous margins ranging from 75.2 to 91.8% at T0, and decreasing to values ranging from 21.3 to 73.9% at T3. Both composite systems and layering protocols had a significant influence on marginal adaptation of the restorations, with statistical associations depending on the restoration group and the timepoint considered. Defective margins in enamel were all of a cohesive nature with micro-fractures, while in dentin, interfacial gaps were the main defect observed. ***Conclusions/Clinical significance:*** The present results highlighted significant degradation of marginal adaption after long-term in vitro fatigue test using materials even with high-viscosity conventional resin composites, applied with a proper layering approach in medium–large sub-gingival cavities. While no significant differences were observed at the dentin cervical margins, there was a tendency for better adaptation at the enamel margin when using a higher modulus material with a multi-layered technique.

## 1. Introduction

Bulkfill resin composites reached a good level of acceptance by clinicians due to the reduced operative time in placing class I and II direct restorations [1]. Their widespread use is also supported by a rather abundant in vitro research substantiating interesting physico-chemical features such as increased polymerization depth and reduced volumetric polymerization shrinkage and associated shrinkage stresses [2,3]. Bulkfill resin composites also show adequate polymerization through layer thicknesses up to 4 mm or even more, which is largely superior to conventional direct restorative materials [4,5]. However, regarding the reduced volumetric shrinkage and shrinkage stresses, high-viscosity bulkfill composites actually present lower shrinkage stress as compared to conventional resin composites, while flowable bulkfill technology exhibits comparable volumetric shrinkage stress to their conventional counterparts [6,7,8]. The changes in bulkfill resin composite matrix structure play an important role in reducing shrinkage stress [9,10,11,12].

When the short- to medium-term clinical performances of both restorative and flowable bulkfill resin composites were tested against traditional layered class I and II direct restorations, results proved satisfactory [13,14,15,16]. Only a few clinical studies are available to compare bulkfill composite restorations to conventional ones with a 5-year follow-up or more; those available reported comparable performance so far. As stated by the majority of authors, such findings must be considered with caution due to the dominant impact of short-term studies and the weak design and bias risk of many studies as well. Cavity design and size are usually not reported, representing another limitation for fully relevant data interpretation. While we have strong evidence about the satisfactory long-term performance (up to 20 years) of conventional resin composites [17,18,19,20], the long-term behavior of bulkfill resins is largely unknown. In particular, the possible impact of a low elastic modulus on the tooth–restoration response to highly functional and parafunctional load conditions is to be properly investigated; the later consideration would be of major significance for biomechanically weakened teeth (i.e., extensive cavities, cracked and non-vital teeth).

Despite the importance and significance of clinical research, numerous confounding factors still limit its potential to identify individual influential factors on restorations’ performance as the observed outcome sums the impact of all identified but also non-identified variables [21,22,23]. The strength of conclusions based on clinical trials would indeed probably benefit from improved categorization in tooth anatomy, cavity size and geometry, masticatory function, patient’s socio-economic environment and control of risk factors, which shall then be analyzed using a multivariate statistical methodology. Fulfilling such an aim is a difficult, time-consuming and expensive task, requiring substantial resources, a large-scale sample size and a long follow-up period. For these reasons, the use of appropriate in vitro protocols is useful to attempt to provide clinicians with guidance while these valuable long-term clinical data are collected. Such protocols present the advantage of allowing standardization of samples as well as restorative and testing conditions for adhesive restorations. The simulation of strains leading to clinical aging and restoration failures is an important asset of such laboratory testing [24,25,26,27,28,29]. Despite the well-documented advantages of in vitro thermomechanical simulation, there is still missing consensus regarding the recommended number of fatigue cycles, their frequency, force profile and loading conditions to optimally simulate in vivo conditions. While most studies apply low masticatory forces (e.g., 45–50 N which is considered a “physiological” strain level in molars) [30,31,32], it would be interesting to also evaluate the impact of higher forces generated for instance during bruxism and clenching [30,31,32] due to their high prevalence in modern populations [33,34,35]. An additional concern frequently mentioned regarding the aforementioned protocols is the use of low cycling numbers which emulate only a short period of clinical function (e.g., 100,000 cycles, which represents less than one year of in vivo function) whereas it would be greatly beneficial to use this methodology to pre-assess medium- to long-term clinical use, which was taken into consideration in the present trial. 

In view of the debate around the correlation between in vivo success or failure rates of adhesive class II restorations and their in vitro performance in thermomechanical simulations (fatigue) [36,37,38], a conclusion could not be made due to the impact of the many aforementioned clinical confounding factors. 

Likewise, we need to gain an understanding about the interpretation and meaning of fatigue tests to strengthen their clinical relevance and likely expand their use for the pre-clinical testing of new materials and restorative techniques. To this aim, we may gain understanding about the number of cycles and forces to be applied for a discriminating comparison between various materials, adhesives and restorative protocols.

The aim of this in vitro study was to test the null hypothesis that the restorative material (conventional nano-hybrid composite on the one hand, high-viscosity and flowable bulkfill composites on the other hand) and application protocol (bulkfilling, single- or multi-layering techniques) do not influence the marginal adaptation of medium–large supra- and sub-gingival class II restorations after staged, extended simulated thermo-occlusal stressing.

## 2. Materials and Method

The methodology was similar to a previously published work by our group [39].

### 2.1. Basic Specimen Preparation and Group Distribution (New) 

In this study, restorations were placed on human third molars, freshly extracted. Only teeth with complete root formation and without carious lesions or extraction-related lesions in proximity to the future restoration margins were selected. Extracted teeth were stored in saline solution at low temperature (5 °C) until the experiment.

Each tooth specimen was stabilized with a small increment of light-curing composite on a brass holder (I.S.T. Innovative Technology GmbH, Ebnat-Kappel, Switzerland) using a custom-made positioning device to control the central and horizontal axis before embedding the root(s) up to 3 mm below the cementoenamel junction (CEJ), using a self-curing acrylic resin (Technovit 4071; Kulzer, Hanau, Germany).

To improve restoration adaption and facilitate material placement, a silicone index of the intact tooth was made before cavity preparation with a putty silicone (Memosil 2, Heraeus Kulzer, Hanau, Germany). The silicone impression was then centrally cut bucco-lingually to obtain two half (mesial and distal) indexes, replacing a matrix.

**Figure 1:**
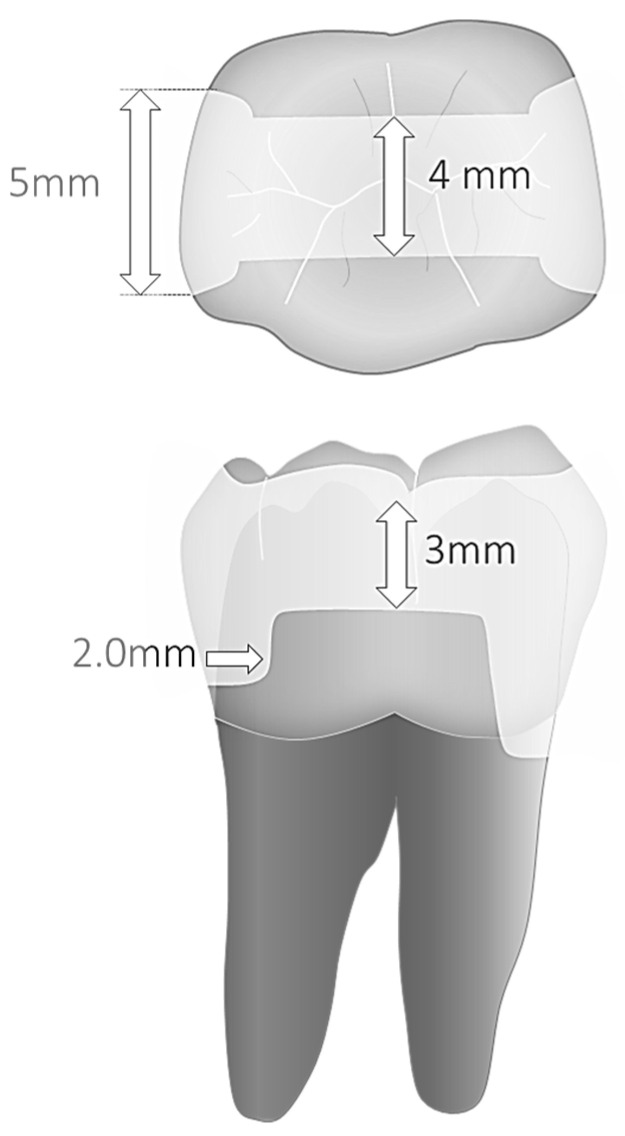
(**top**) Cavity dimensions and (**bottom**) margin sectors under evaluation.

A parallelometer controlling hand-piece movements and ensuring constant cavities’ dimensions and geometry served to prepare Class II cavities (MOD) with parallel walls. Mesially, margins were prepared 1.0 mm above and distally 1 mm below the cementoenamel junction. Cavity preparation and finishing were performed with a microscope at 8–10× magnification. The dimensions of the parallel preparations were 5.0 mm in width and 2.0 mm in depth at the bottom of the proximal box, and 4.0 mm in width and 3.0 mm depth for the occlusal isthmus (Figure 1). A 1.0–1.5 mm bevel was placed in all enamel margins.

The 40 prepared teeth were randomly distributed within the following experimental groups (*n* = 8 samples per group):

- Group 1: **Multilayered** restorations made of conventional nano-hybrid composite—Tetric EvoCeram (Ivoclar, Schaan, Liechtensetein) (control group);
- Group 2: **Single layer** restorations made of high-viscosity bulkfill composite—Tetric EvoCeram Bulk Fill (Ivoclar);
- Group 3: **Trilaminated** restorations made of high-viscosity bulkfill composite—Tetric EvoCeram Bulk Fill;
- Group 4: **Bilaminated** restorations made of one single increment of flowable bulkfill composite—Surefil SDR flow bulkfill (Sirona-Dentsply, Charlotte, NC, USA)—and one occlusal layer of conventional nano-hybrid composite Ceram-X (Sirona-Dentsply);
- Group 5: **Trilaminated** restorations made of two increments of flowable bulkfill composite—Surefil SDR flow bulkfill—and one occlusal layer of conventional nano-hybrid composite Ceram-X.

Add all details of Product/Shade, Batch Number, Composition, Manufacturer are detailed in Table 1.

### 2.2. Restorative Procedures 

#### 2.2.1. Adhesive Procedures

Enamel and dentin surfaces were etched selectively for 30 s and 10 s, respectively, before the application of a three-step etch and rinse adhesive (OptibondFL unidose, Kerr, Brea, CA, USA). Following an abundant rinsing, water was removed with suction prior to primer and bonding application following the manufacturer’s instructions. Bonding was light-cured for 20 s at 1200 mW/cm^2^ irradiance.

#### 2.2.2. Restorative Protocols

The specific protocol and material selection follows the group distribution section:

- Group 1 (control):

Tetric EvoCeram was applied with a multi-layering technique. The proximal areas were restored first, using one cervical, horizontal layer of 1.5 mm, followed by two vertical layers (the last one being about 1.5 to 2 mm width). The remaining occlusal volume (cavity isthmus) was restored with two oblique layers (facial and lingual).

- Groups 2 and 3:

Tetric EvoCeram Bulk Fill was applied in either a single increment to restore the entire cavity (Group 2) or with a trilaminar technique (Group 3), using two horizontal increments in each cavity proximal area and a last occlusal increment to compete the restoration occlusal surface.

- Groups 4 and 5:

SureFil SDR flow was applied either as a single increment base to fill-up the entire cavity volume up to 1.5–2.0 mm below the occlusal surface (Group 4) or with a bilaminated technique comprising a first horizontal increment in each proximal box and a second increment until below the occlusal surface (Group 5); the remaining volume was then filled with a single increment of CeramX Universal restorative material. 

The average depth of sub-gingival cavities was 5 to 6 mm (measured between cervical margin and the proximal ridge level). In groups 2 and 4, the thickness of proximal increments on the dentin side was from 3.5 to a 4.5 mm maximum for the Surefil SDR flow bases, below an occlusal restorative layer of 1.5–2 mm, and 5 to 6 mm for the restorations made of Tetric EvoCeram Bulk Fill. The latter thicknesses remained within the range of published light-curing efficiency for both materials [4]. A calliper served to measure each sample preparation’s depth and increments’ thicknesses. The composite layers for each material were light-cured for 20 s at an irradiance of 1200 MW/cm^2^ with a continuous irradiation mode (Bluephase, Vivadent, Ivoclar, Schaan, Liechtensetein). The silicone index replacing a matrix was coated with an aluminum foil to create a light reflection similar to common sectional metal matrices. The final occlusal increment in all groups was carved/sculpted with hand instruments before a final light curing, to reduce composite polymerization and finishing stresses.

Fine diamond flame and pear-shape burs were used for occlusal surface finishing whereas polishing was performed with silicone points under abundant water spray (Brownies points, Shofu, Tokyo, Japan). Discs of decreasing grain size (Soft Lex Pop On XT, 3M) were used for the finishing and polishing of proximal surfaces. 

### 2.3. Thermomechanical Cycling

A total of 1 week following restoration placement and finishing, the fatigue test was carried out using a well-established protocol [25,26,39,40]. The specimens were exposed to thermomechanical cycling in a chewing simulation machine (Chewing Simulator CS-4.8, SD Mechatronik) in 3 phases: 500,000 cycles twice, followed by 1 million continuous cycles for a total of 2 million cycles. During the 3 phases, an axial loading force of 50 N at a 1.5 Hz frequency using a “low-impact” chewing mode (reducing impact speed, simulating more closely natural chewing pattern) was exerted occlusally together with 3350 thermal cycles between 5 to 55 °C every 500,000 cycles. A 0.5 mm rubber disc was placed below the samples and the chewing simulator sample holder, simulating periodontal ligament stress absorption. 

### 2.4. Specimen Evaluation

The restorations’ margins were cleaned with a brush and fine pumice before a short 5 s surface etching using diluted H_3_PO_4_ gel (Ultraetch, Ultradent, South Jordan, UT, USA); the typical 35–37% gel was diluted with 3 times its volume of distilled water. This procedure was performed before the fatigue test, as well as after completion of the loading phase. The samples’ impression was made with PVS (President light body, Coltenewhaledent, Altstetten, Switzerland) and gold sputtered epoxy resin replicas produced for SEM evaluation (Epofix; Struers, Copenhagen, Denmark). The proximal tooth–restoration interface was then analyzed quantitatively by scanning electron microscopy (SEM) (Sigma 300VP; Zeiss, Gina, Germany) by means of a well-standardized evaluation method [24,40]: continuity (defect free), or for defective margins, overfilling, underfilling, marginal opening, marginal restoration or tooth fracture were the evaluation criteria employed in this study. The following segments were evaluated: proximal, vertical enamel margins (assessed individually on both restoration sides), cervical enamel (restoration side above CEJ) and cervical dentin margin (restoration side below the CEJ) (Figure 1). Occlusal margins were not assessed. Observations were made at a standard 200× magnification. Whenever necessary for the assessment accuracy, higher magnifications were used. 

**Figure 2:**
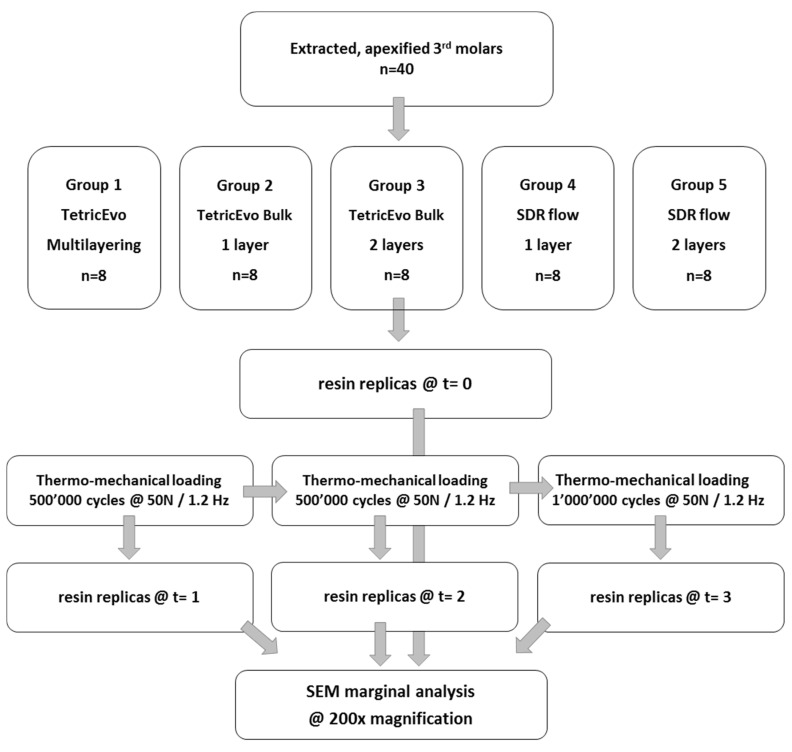
Study workflow.

Results of marginal adaptation before and after the loading phases were expressed as percentages of “continuity”. Percentages were calculated as the ratio between the cumulated distance of all segments with closed, continuous margins and the overall length of the 4 specific margin areas under evaluation (approximal enamel, cervical enamel and cervical dentin). Figure 1 illustrates cavity dimensions and margin sectors under evaluation, while Figure 2 summarizes the study workflow.

All results of the SEM analysis were submitted to a parametric statistical analysis. Repeated measures ANOVA followed by Fisher’s LSD (Least Significant Difference) post hoc tests served for comparing inter-group marginal adaptation percentages (before and after the loading test) and intra-group marginal adaptation percentages, between the pre- and post-loading conditions. The validity of the normality and homoscedasticity assumptions for the repeated measures ANOVA have been checked by means of the Shapiro–Wilk test and Bartlett’s Chi-Square. The sample size (8 per group) was initially assessed according to the existing literature [40,41]. During and after the experiment, the overall power of the test was monitored to evaluate sample size adequacy. The overall power of the repeated measures ANOVA was above 0.7.

## 3. Results

**Figure 3:**
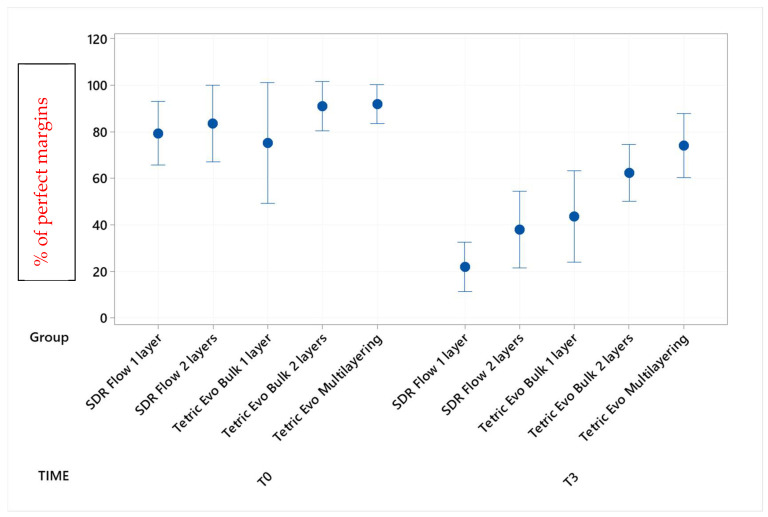
Continuous adaptation of approximal enamel margins (supra-gingival restoration side) at T0 and T3.

**Figure 4:**
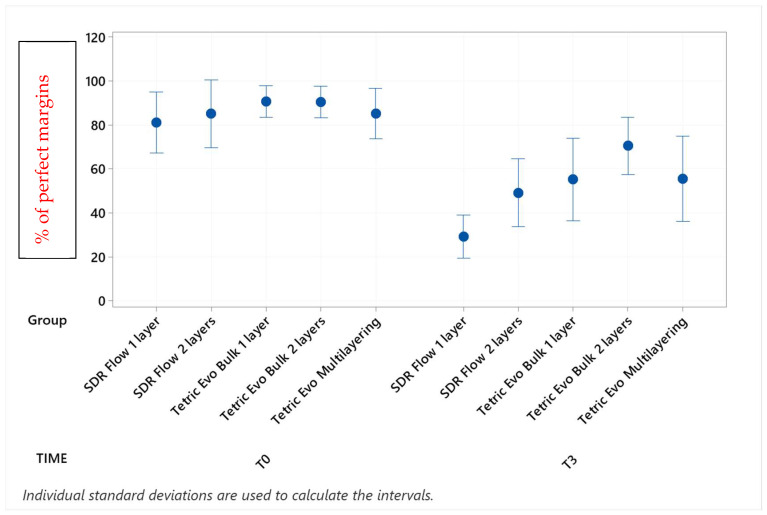
Continuous adaptation of approximal enamel margins (sub-gingival restoration side) at T0 and T3.

**Figure 5:**
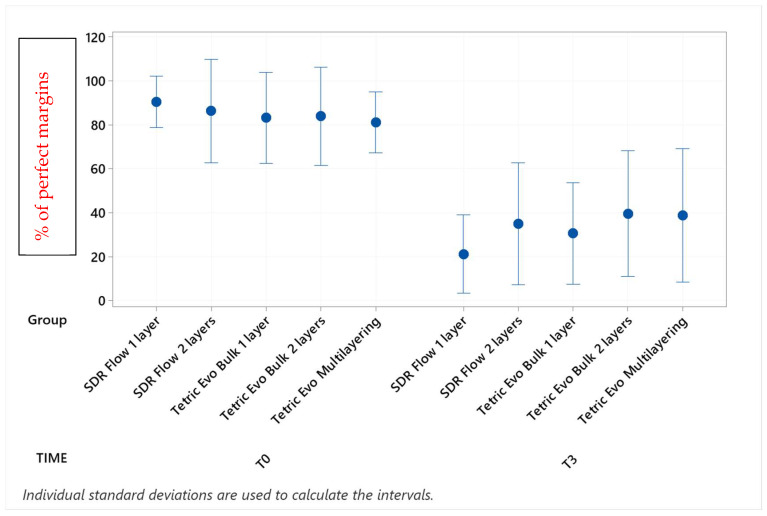
Continuous adaptation of cervical enamel margins (supra-gingival side) at T0 and T3.

**Figure 6:**
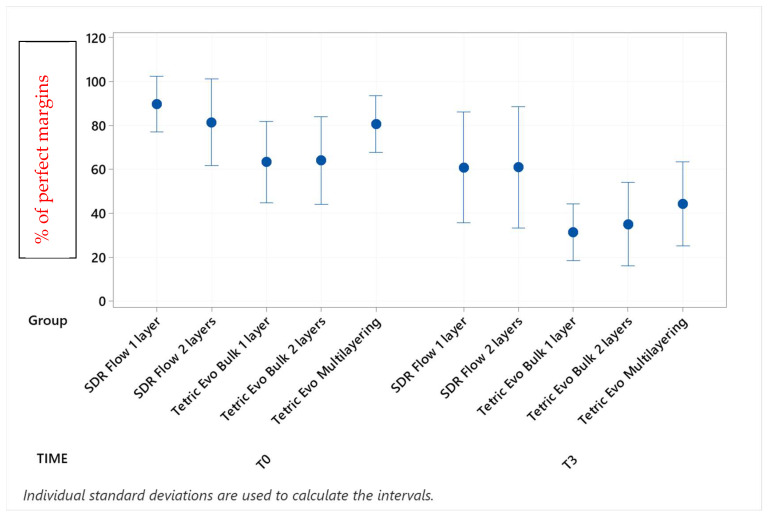
Continuous adaptation of cervical dentin margins at T0 and T3.

Marginal adaptation values before and after the three phases of thermomechanical loading with their statistical analyses are shown in Table 2, Table 3, Table 4 and Table 5 (post hoc groupings) and Figure 3, Figure 4, Figure 5 and Figure 6 (95% interval plots). For the sake of brevity, the tables and figures represent groupings in the T0-T3 comparison only, although the analyses were conducted with repeated measurements at T1 and T2 timepoints also.

Before thermomechanical loading (T0), the values of continuous enamel adaptation varied from 91.8% (Tetric EvoCeram Multilayering) to 75.2% (Tetric EvoCeram Bulk Fill 1 layer) in proximal margins of the supra-gingival restoration side, from 90.7% (Tetric EvoCeram Bulk Fill 1 layer) to 85.1% (SDRflow 2 layers) in proximal margins of the sub-gingival restoration side and from 95.4% (SDRflow 1 layer) to 81.2% (Tetric EvoCeram multilayering) at the cervical enamel margins. The initial values for continuous cervical dentin adaptation varied from 89.8% (SDRflow 1 layer) to 63.4% (Tetric EvoCeram Bulk Fill 1 layer). There was no statistical difference between products at T0 for the four considered interfaces areas.

Following thermomechanical loading, the values of continuous enamel adaptation on the supra-gingival restoration side varied from 84.1% (Tetric EvoCeram Multilayering) to 44.3% (SDRflow 1layer) at T1, from 81.4% (Tetric Evoceram Multilayering) to 35.5% (SDRflow 1 layer) at T2 and from 74.0% (Tetric EvoCeram Multilayering) to 21.9% (SDRflow 1 layer) at T3. On the sub-gingival restorations side, continuous enamel adaptation varied from 82.7% (Tetric EvoCeram Bulk Fill 2 layers) to 45.4% (SDRFlow 1 layer) at T1, from 79.4% (Tetric EvoCeram Bulk Fill 2 layers) to 34.9% (SDRFlow 1 layer) at T2 and from 70.5% (Tetric EvoCeram Bulk Fill 2 layers) to 29.3% (SDRFlow 1 layer) at T3. The cervical enamel continuous adaption varied from 66.4% (Tetric EvoCeram Bulk Fill 2 layers) to 54.8% (SDRFlow 2 layers) at T1, from 55.4% (Tetric EvoCeram Multilayering) to 30.1% (SDRFlow 1 layer) at T2 and from 39.6% (Tetric EvoCeram Bulk Fill 2 layers) to 21.3% (SDRFlow 1 layer) at T3. The dentin continuous cervical adaptation varied from 79.1% (SDRFlow 1 layer) to 54.8% (Tetric EvoCeram Bulk Fill 1 layer) at T1, from 71.4% (SDRFlow 1 layer) to 38.0% (Tetric EvoCeram Bulk Fill 1 layer) at T2 and from 61.0% (SDRFlow 2 layers) to 31.4% (Tetric EvoCeram Bulk Fill 1 layer) at T3. 

Defective margins in enamel were all of cohesive nature with micro-fractures while in dentin, gaps were the main defects observed. As shown by the above data, thermomechanical loading induced a drop of all continuous adaptation values within the four segments under evaluation, and the statistical associations varied between products, layering strategies and experimental setup. Table 2, Table 3, Table 4 and Table 5 detail the continuous adaptation means and significant differences for the four margin sections under evaluation over the three test phases. 

TetricEvo multilayering showed non-significant adaptation differences over the whole fatigue test for approximal enamel margins on the supra-gingival side while there was a significant drop for the other three margin sections with increasing numbers of fatigue cycles. Both Tetric EvoCeram Bulk Fill groups showed a significant adaptation decrease in the three enamel sections and cervical dentin with increasing fatigue cycles. SDR Flow groups also showed a significant marginal degradation in the three enamel sections while there was no significant change in dentin cervical adaptation for SDR Flow 2 layers and only a significant difference for SDR Flow 1 layer between T0 and T3.

When comparing experimental conditions at T3, there were no significant differences between the various material and layering combinations for cervical dentin (Table 5) and enamel (Table 4) margins. On the contrary, significant differences were observed for approximal enamel margins on both supra- and sub-gingival restoration sides, with Tetric Evo Multilayering and Tetric Evo Bulk 2 layers performing significantly better, and SDR Flow performing significantly worse (Table 2 and Table 3).

**Figure 7:**
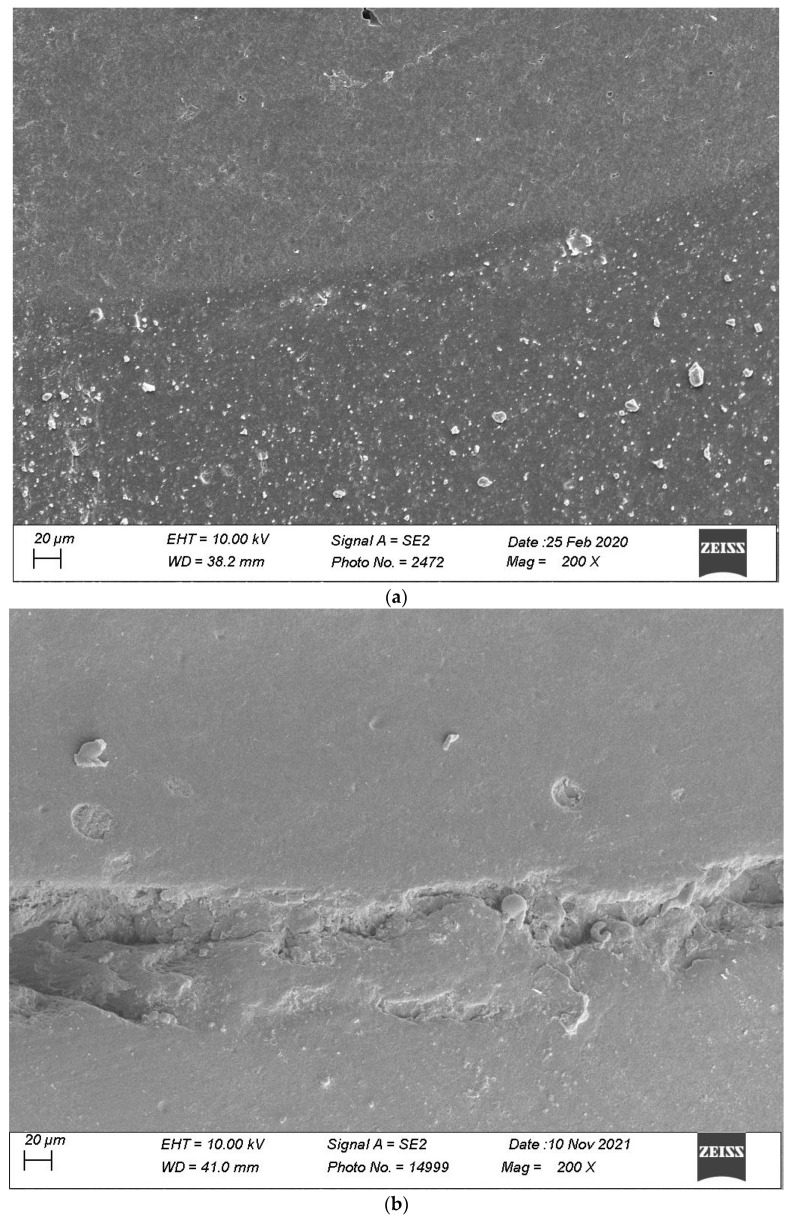
(**a**,**b**) shows, respectively, SEM images’ margins at T0 and margins at T3 of Group 1.

**Figure 8:**
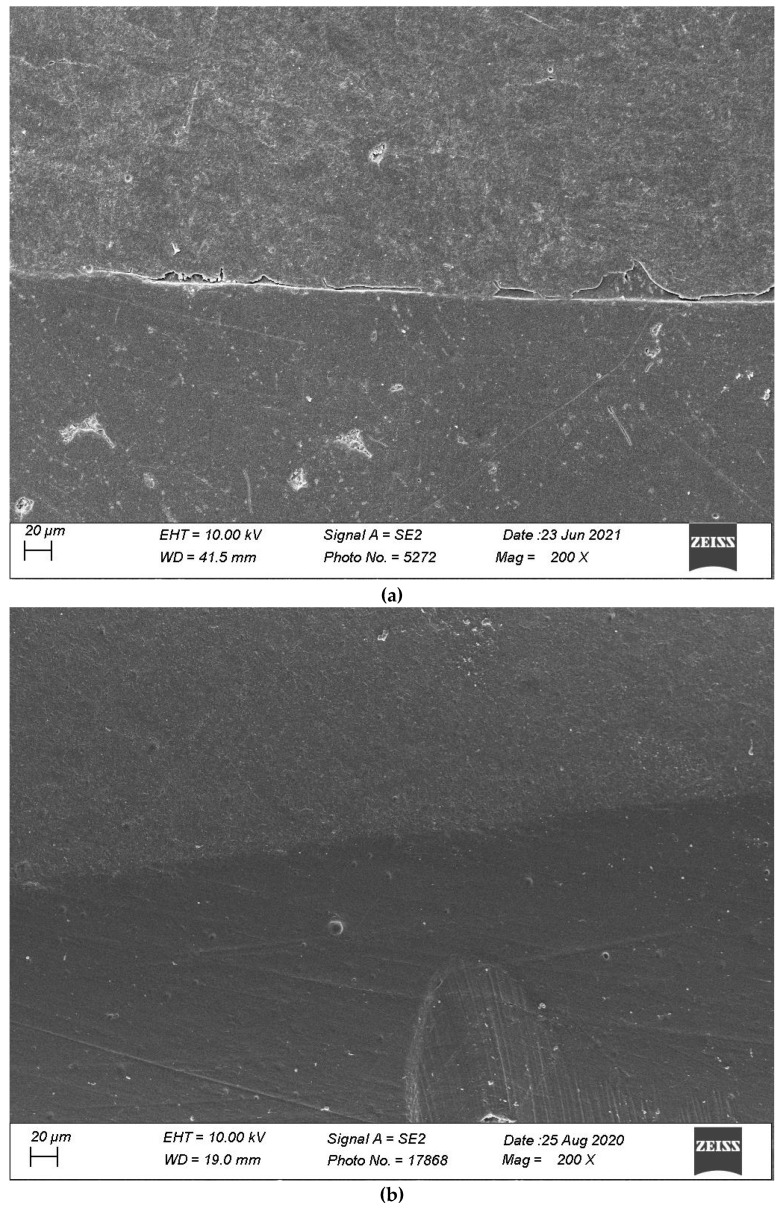
(**a**,**b**) shows, respectively, SEM images’ margins at T0 and margins at T3 of Group 2.

**Figure 9:**
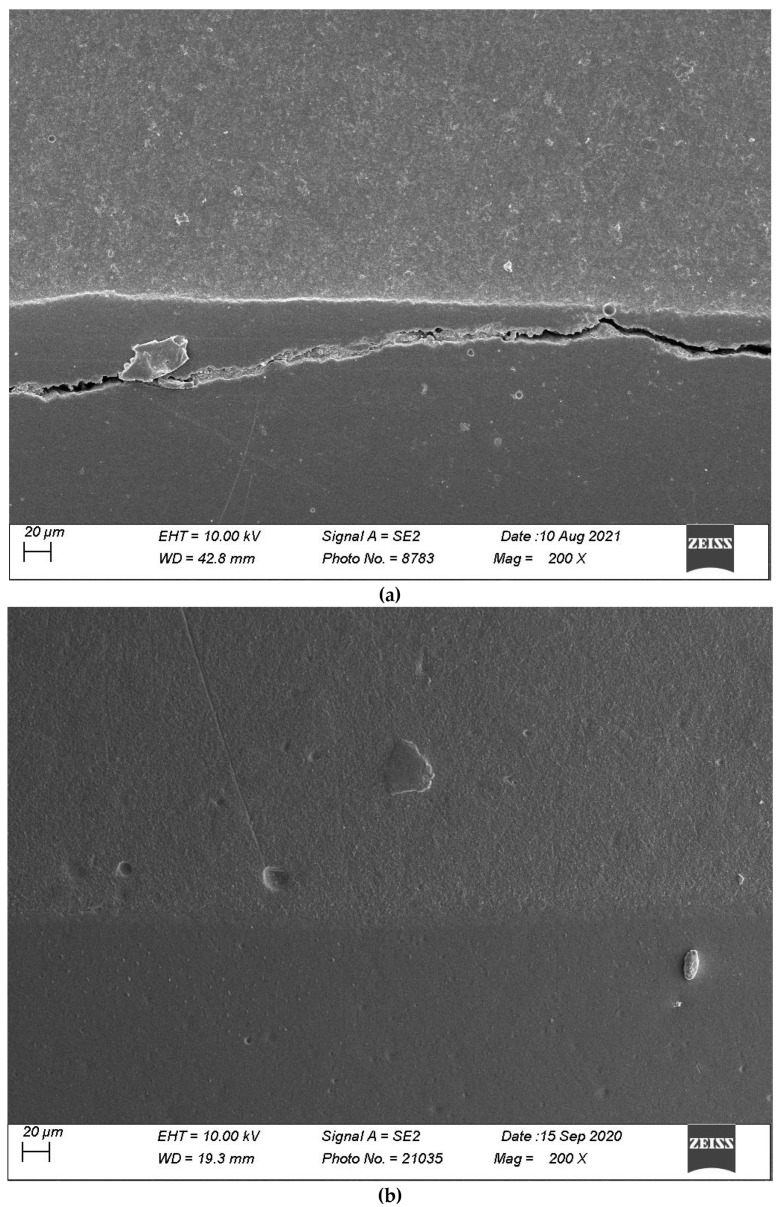
(**a**,**b**) shows, respectively, SEM images’ margins at T0 and margins at T3 of Group 3.

**Figure 10:**
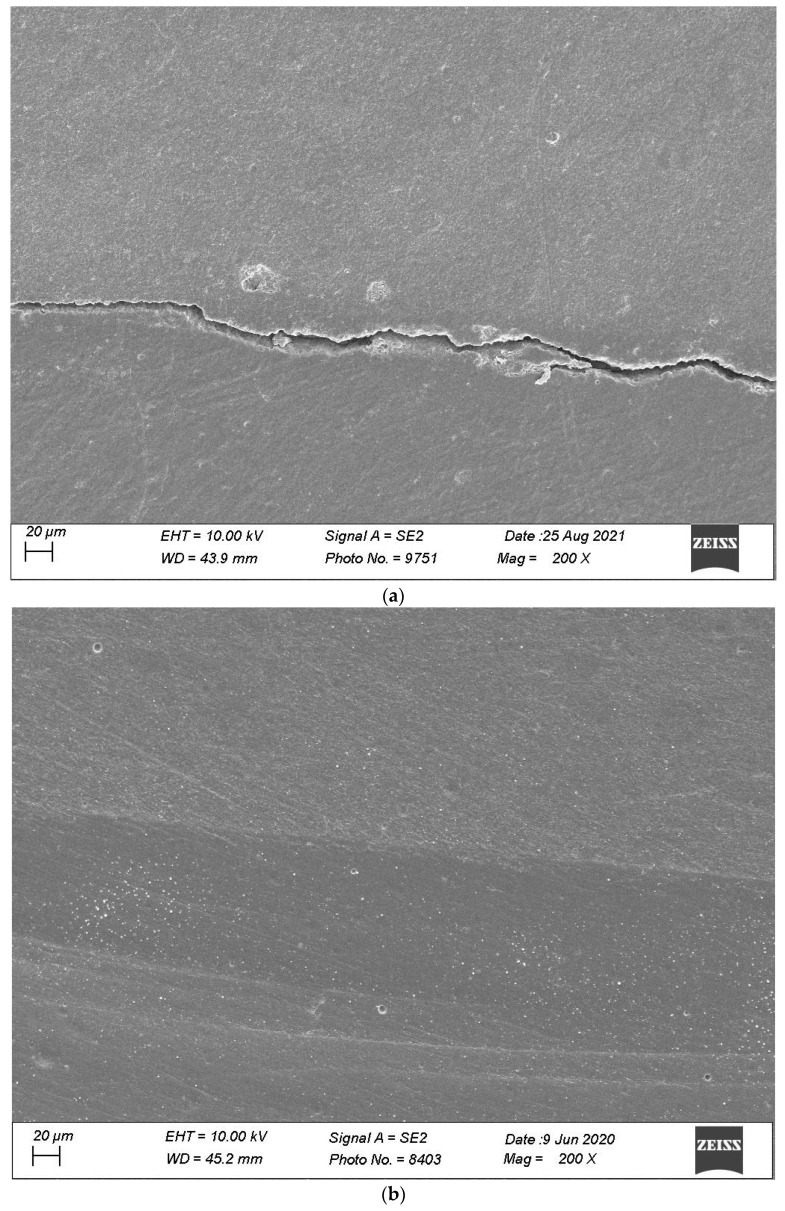
(**a**,**b**) shows, respectively, SEM images’ margins at T0 and margins at T3 of Group 4.

**Figure 11:**
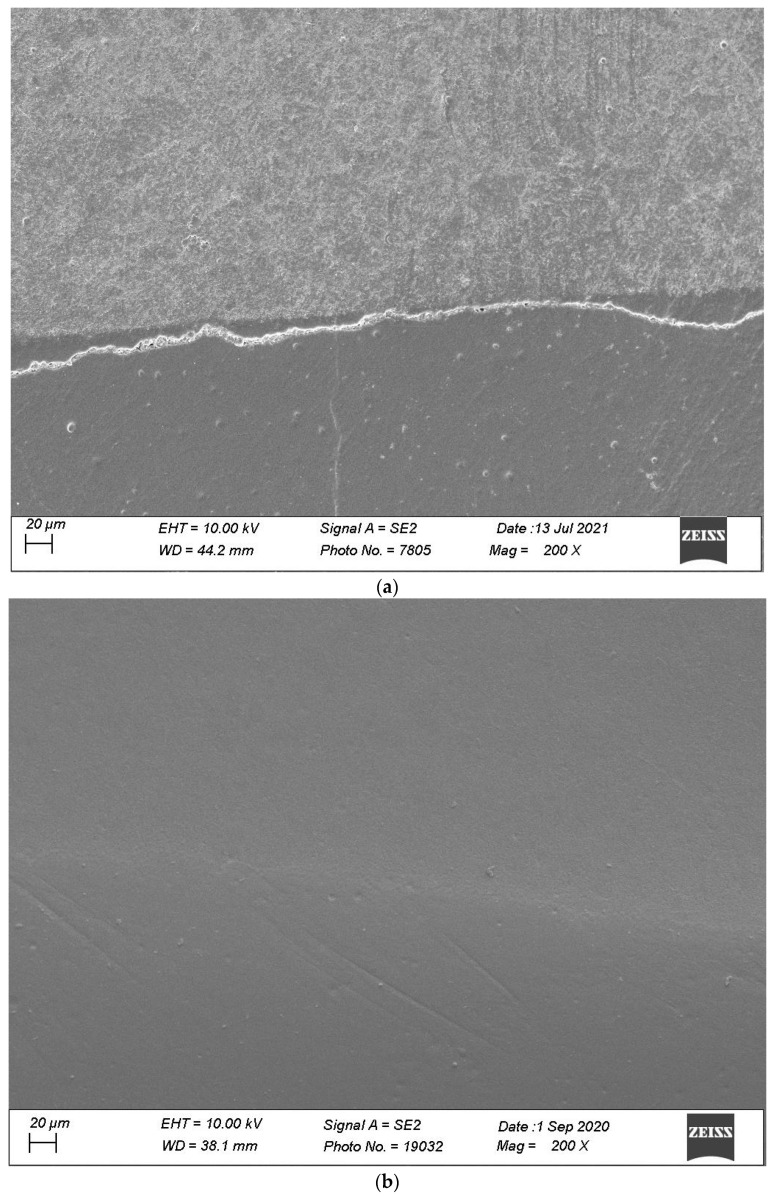
(**a**,**b**) shows, respectively, SEM images’ margins at T0 and margins at T3 of Group 5.

Figure 7, Figure 8, Figure 9, Figure 10 and Figure 11 show, respectively, SEM images of continuous margins and defective margins, as evaluated in the study. 

## 4. Discussion

The study’s null hypothesis was rejected, as composite systems and layering protocols both had a significant influence on marginal adaptation of medium–large class II restoration with supra- and sub-gingival cervical margins following various periods of thermomechanical loading. 

### 4.1. Impact of Thermomechanical Loading

Marginal adaptation is an important aspect when considering success or failure of any restoration type as it controls the necessary biological seal between restorative materials and surrounding tooth tissues. Restoration failure involves repeated thermal, chemical and mechanical strains [42,43]. Therefore, simulation of intra-oral restoration aging in setups mimicking physiological masticatory function and thermal cycling in a moist environment have proven to be a useful in vitro testing approach [28,29]. There is, however, still controversy and limited evidence with regard to load intensity and number of applied cycles needed to emulate short-, medium- or long-term in vivo behaviour within physiological or parafunctional environments. The few attempts made to correlate outcomes of in vivo clinical trials and in vitro marginal adaptation experiments failed to reveal a clear relationship [43,44]. More recently, however, the predictive value of early in vivo marginal adaptation on long-term restoration behaviour could be highlighted [45]. In the present study, marginal adaptation declined over the successive loading phases, with a marked change between pre-loading conditions (T0) and 1 million cycles (T2) or between a half-million (T1) and 2 million cycles (T3), suggesting that a low number of loading cycles (i.e., 100,000 cycles) likely reduces the discriminative power of this testing method. Considering the number of yearly masticatory cycles in humans range from 250 to 300,000 [25,46], this study emulated the potential impact of 6.5 to 8 years of clinical service. The significantly higher number of cycles in the present work has therefore certainly increased the predictive value of the preservation of marginal adaptation quality over time. Another important variable when considering the preservation of marginal adaptation over time is the impact of cycling load. In the present study, 50 N of occlusal load was applied to the samples, which is consistent with most similar fatigue studies. This load value lies in the lowest range of recorded biting forces per tooth contact in molars and premolars (10–885 MPa) [30,31,32,47,48,49], which corresponds to maximal physiological biting forces. In a parafunctional environment, clenching or bruxism forces will be of much higher magnitude and considering their significant prevalence [33,34,35], using higher load conditions will be of interest to expand the relevance of such testing approach. Therefore, a subsequent work is ongoing to investigate the impact of higher loading forces with the same restorative strategies and methodology. 

A last parameter of importance with regard to the present methodology is the threshold of marginal adaptation (in %) below which an increased risk of clinical failure is expected (simulated service life). Some studies reported that marginal and internal adaptation are closely correlated and that interfacial defects, in particular gaps, progress toward pulpal wall and cavity floor with an increasing extent of marginal defects [29,50]. The analysis and interpretation of those data suggested that marginal adaptation values below 50% relate to sub-optimal restoration quality, in particular at the dentin level. For example, in similar testing conditions, with different thermomechanical equipment, indirect restorations presented much higher percentages of continuous adaptation at dentin margins (above 90% for the best groups) [50,51,52], a finding correlated with a reduced risk of failures clinically [45]. 

### 4.2. Layering Protocols

The traditional layering protocol using Tetric EvoCeram provided satisfactory adaptation in the three enamel sections, overall comparable to the Tetric EvoCeram Bulkfill. In dentin, both Tetric EvoCeram products presented a marked drop of continuous adaptation values along the three loading phases, multi-layered Tetric EvoCeram restorations having, however, the best score at the end of the three thermomechanical loading phases. 

Tetric EvoCeram Bulk fill applied in two layers performed overall better than when using the single layer approach in proximal enamel margins for both supra- and sub-gingival restoration sides. In cervical enamel and dentin margins, despite inferior percentages of continuous adaptation with a single layer approach over the four observation periods, the difference was not significantly different. SDR flow applied in two layers also showed improved proximal enamel adaptation as compared to a single-layer approach after thermomechanical loading in the supra- (T1) and sub-gingival restoration sides (T1 to T3). However, in cervical enamel or dentin margins, the number of SDR flow layers did not significantly affect the marginal adaption before or after loading, although overall results support the interest of applying a simple layering approach (i.e., bilaminar) to enhance restoration adaptation in large and deep proximal cavities when using bulkfill resin composites. The latter results are in partial agreement with another in vitro testing showing reduced internal gap formation, albeit without mechanical loading [40]. The impact of simplified layering using bulkfill materials was, however, not evaluated using similar thermomechanical fatigue testing approach. 

### 4.3. Products and Inter-Group Comparisons

The inter-group comparisons showed that both products and restorative protocols had a significant effect on restoration adaptation at the dentin margins already before loading and at both dentin and enamel margins following thermomechanical loading. However, despite inter-group differences being present, the effect of simulated aging (T0 to T3) was much larger than the inter-group differences.

Regarding the pre-loading adaptation, all tested products and layering protocols demonstrated a high percentage of defect-free margins, confirming that the used materials and layering protocols were able to appropriately manage polymerization shrinkage stresses in the short term. For some materials, some specific features were implemented to control shrinkage stresses, for example, Tetric EvoCeram Bulk fill which contains a shrinkage stress absorber in the form of a patented, special filler (prepolymer). The elastic modulus of the latter is relatively low at 10 GPa (by comparison, the modulus of elasticity of glass fillers is approximately 71 GPa). This filler supposedly acts like a microscopic spring and absorbs the stresses generated during the photopolymerization. Another example is the higher deformability (lower E-modulus) of SDR bulkfill flowable resin composite due, among others, to the presence of plasticisers. Such a low E-modulus has been associated with a “stress-absorbing” potential effect before loading, with a tendency for less gap formation as compared to high-viscosity bulkfill material applied in one or two layers; these findings are in line with previous results [53]. 

Post-loading, layered restorations with conventional composite or high-viscosity bulkfill composites performed better than the flowable bulkfill composite in proximal enamel margins. In cervical enamel, the single-layer bulkfill flow approach also showed a higher proportion of microcracks, in particular after extended loading. The percentages of microcracks measured in this study are in line with published data comparing enamel adaption of restorative and flowable bulkfill materials [54]. Conversely, in cervical dentin, no significant differences could be observed between the different groups. A previous study also highlighted a superior marginal adaptation to enamel after thermomechanical loading for composites having a higher elastic modulus (close to dentin), but failed to show an influence of this variable on dentin adaptation [55]. 

Restorative procedures and function generate rather complex stress patterns, with various spatial distribution within tooth structures, the restoration, and the tooth–restoration interfaces [56,57,58]. Thermomechanical interactions also involve specific macro- and micro-deformation phenomena within both restorative materials and tooth tissues; considering their dissimilar structure and stiffness, adaptation to enamel and dentin was then expected to evolve differently before and after thermomechanical fatigue. One identified key factor is the difference in tested materials’ stiffness (E-modulus) and volumetric shrinkage. Independently from their reduced polymerisation shrinkage, the higher E-modulus of high-viscosity composites (Tetric EvoCeram and EvoCeram Bulk fill) [9,10,11,12] may have played a positive role in limiting differential deformations upon thermomechanical fatigue between the restoration and tooth tissues, thereby limiting the development of microcracks at bevelled enamel margins. With thin layers of flowable bulkfill composite covering enamel bevels, deformation under loading provoked, in contrast, higher percentages of micro-cracks. Interestingly, restorations made with SDR flow exhibited the best and worst enamel cervical adaptation, respectively, before and after thermomechanical loading, supporting the rationale of load-induced micro-deformation and cohesive failure paired to the low enamel tensile strength. Fundamentally, the ratio between tissue ultimate tensile strength (low for enamel and high for dentin) [59,60,61] and maximal adhesive bonding strength governs the consequences on dental tissues of interfacial stresses and eventual failure types. Then, overall, polymerization, finishing and thermomechanical strains induce gap formation at the restoration–dentin interface or micro-cracks at enamel margins, as confirmed in the present study. 

Despite the interesting trends highlighted in this simulated aging in vitro study, some limitations have to be underlined. Even if important efforts were made in order to best simulate clinical conditions, the results obtained have to be verified in long-term prospective clinical trials. 

Finally, as mentioned in the introduction, most studies investigating accelerated aging of composite restorations apply low masticatory—physiological—forces, as was the case in the present work. Therefore, in order to evaluate how the investigated materials and filling strategies would perform under non-physiological—parafunctional—loading, a second work will follow, where the present methodology will be repeated but using higher loads.

## 5. Conclusions

The present results highlighted significant degradation of marginal adaption after long-term in vitro fatigue test using materials with high-viscosity such as conventional or modern bulkfill resin composites, even with proper layering approach in medium–large sub-gingival cavities. While no significant differences were observed at the dentin cervical margins, there was a tendency for better adaptation at the enamel margin when using a higher modulus material with a multi-layered technique. The null study hypothesis was therefore rejected, confirming an influence of both materials and layering methods on tooth–restoration interfaces, albeit to a lesser extent compared to the effect of loading cycles.

## Figures and Tables

**Table 1:** Products under investigation.

Product/Shade (Abbreviation)	Batch Number	Composition	Manufacturer
OptiBond FL (adhesive)	8626023	Primer: HEMA, GPDM, MMEP, ethanol (20–25%), water, initiators Adhesive: uncured methacrylate Ester, TEGDMA, Ytterbium trifluoride, inert mineral fillers, photoinitiators, stabilizers	Kerr Corpopration, Orange, CA, USA
Tetric EvoCeram B2 (TEC)	W82536	Matrix: Bis-GMA, UDMA, Ethoxylated Bis A Dimethacrylate Fillers: Barium glass filler, Ytterbium trifluoride, mixed oxide, pre-polymers, fumed silica Photoinitiator (CQ)	Ivoclar, Schaan, Liechtensetein
Tetric EvoCeram Bulkfill (TECB)	Y46174	Matrix: Bis-GMA, UDMA, Ethoxylated Bis A Dimethacrylate Fillers: Barium glass filler, Ytterbium trifluoride, mixed oxide, pre-polymers, fumed silica Photoinitiators (CQ and Ivocerine)	Ivoclar, Schaan, Liechtensetein
SDR Flow Univ (SDR)	00043289	Matrix: modified urethane dimethacrylate resin, ethoxylated bisphenol-A dimethacrylate, triethyleneglycol dimethacrylate, camphorquinone, butylated hydroxyl toluene, UV stabilizer, titanium oxide, iron oxide pigments Filler: barium-alumino-fluoro-borosilicate glass, strontium alumino-fluoro-silicate glass	Dentsply DeTrey, Constance, Germany

**Table 2:** Continuous adaptation of approximal enamel margins (supra-gingival restoration side).

Group × Timepoint	% Mean	SD	Grouping		
Tetric Evo Multilayering T0	91.8	9.9	A		
Tetric Evo Bulk 2 layers T0	90.9	12.8	A		
SDR Flow 2 layers T0	83.4	19.7	A		
SDR Flow 1 layer T0	79.2	16.3	A		
Tetric Evo Bulk 1 layer T0	75.2	31.0	A		
Tetric Evo Multilayering T3	73.9	16.4	A		
Tetric Evo Bulk 2 layers T3	62.2	14.6	A	B	
Tetric Evo Bulk 1 layer T3	43.6	23.4		B	C
SDR Flow 2 layers T3	37.9	19.7		B	C
SDR Flow 1 layer T3	21.9	12.6			C

**Table 3:** Continuous adaptation of approximal enamel margins (sub-gingival restoration side).

Group × Timepoint	% Mean	SD	Grouping			
Tetric Evo Bulk 1 layer T0	90.7	8.5	A			
Tetric Evo Bulk 2 layers T0	90.5	8.5	A			
Tetric Evo Multilayering T0	85.2	13.7	A			
SDR Flow 2 layers T0	85.1	18.5	A			
SDR Flow 1 layer T0	81.2	16.5	A	B		
Tetric Evo Bulk 2 layers T3	70.5	15.6	A	B	C	
Tetric Evo Multilayering T3	55.5	23.1		B	C	D
Tetric Evo Bulk 1 layer T3	55.3	22.5		B	C	D
SDR Flow 2 layers T3	49.2	18.5			C	D
SDR Flow 1 layer T3	29.3	11.7				D

**Table 4:** Continuous adaptation of cervical enamel margins (supra-gingival side).

Group × Timepoint	% Mean	SD	Grouping			
SDR Flow 1 layer T0	90.5	14.1	A			
SDR Flow 2 layers T0	86.4	28.2	A			
Tetric Evo Bulk 2 layers T0	83.9	26.8	A	B		
Tetric Evo Bulk 1 layer T0	83.2	24.6	A	B	C	
Tetric Evo Multilayering T0	81.2	16.5	A	B	C	
Tetric Evo Bulk 2 layers T3	39.6	34.2		B	C	D
Tetric Evo Multilayering T3	38.9	36.2			C	D
SDR Flow 2 layers T3	35.0	33.2				D
Tetric Evo Bulk 1 layer T3	30.6	27.5				D
SDR Flow 1 layer T3	21.3	21.2				D

**Table 5:** Continuous adaptation of cervical dentin margins.

Group × Timepoint	% Mean	SD	Grouping		
SDR Flow 1 layer T0	89.8	15.1	A		
SDR Flow 2 layers T0	81.4	23.5	A	B	
Tetric Evo Multilayering T0	80.6	15.5	A	B	
Tetric Evo Bulk 2 layers T0	64.1	23.8	A	B	C
Tetric Evo Bulk 1 layer T0	63.4	22.1	A	B	C
SDR Flow 2 layers T3	61.0	32.9	A	B	C
SDR Flow 1 layer T3	61.0	30.0	A	B	C
Tetric Evo Multilayering T3	44.4	22.9		B	C
Tetric Evo Bulk 2 layers T3	35.1	22.7			C
Tetric Evo Bulk 1 layer T3	31.4	15.4			C

## Data Availability

Not applicable.
